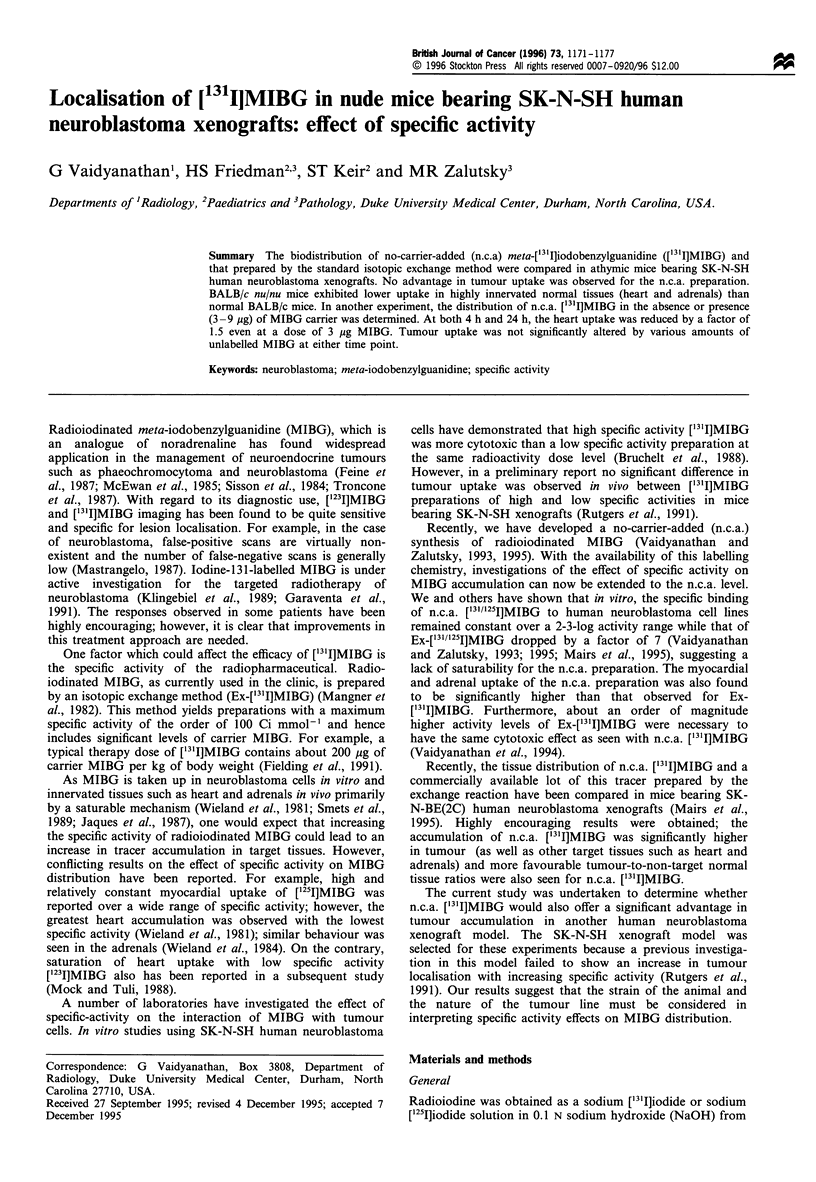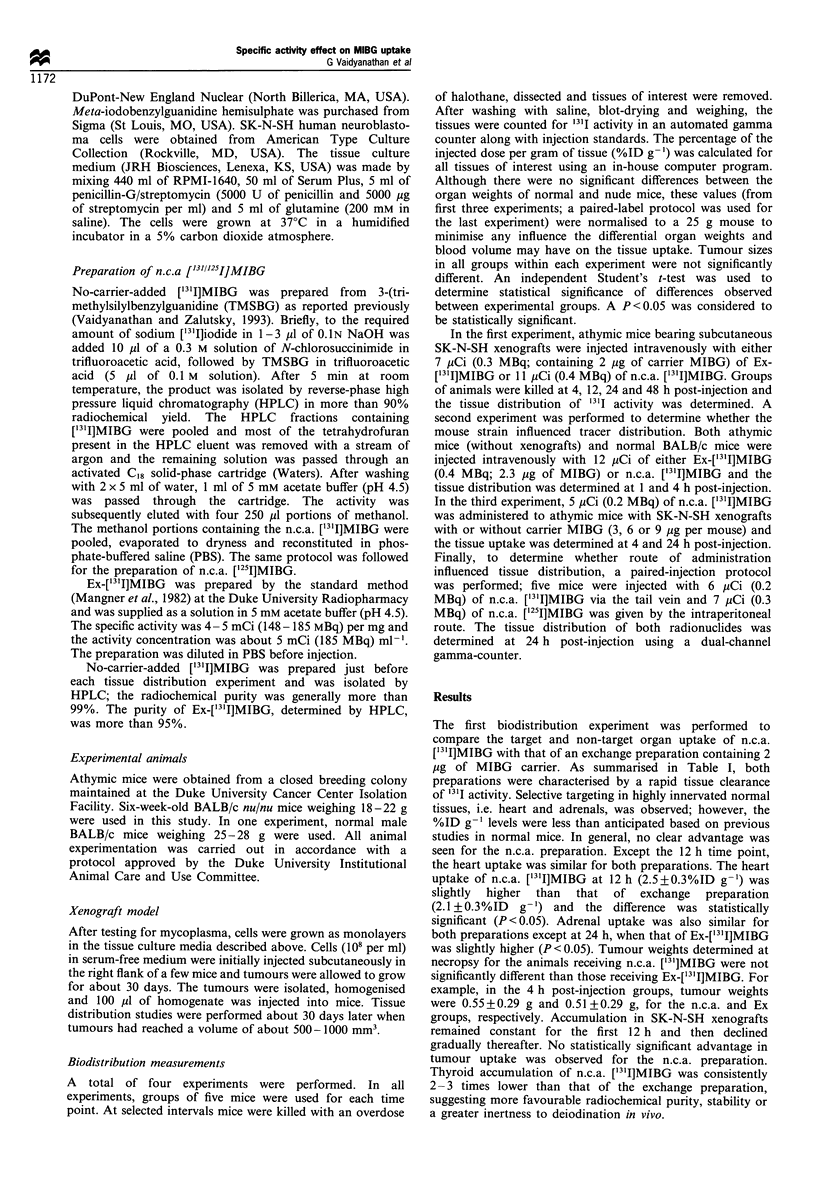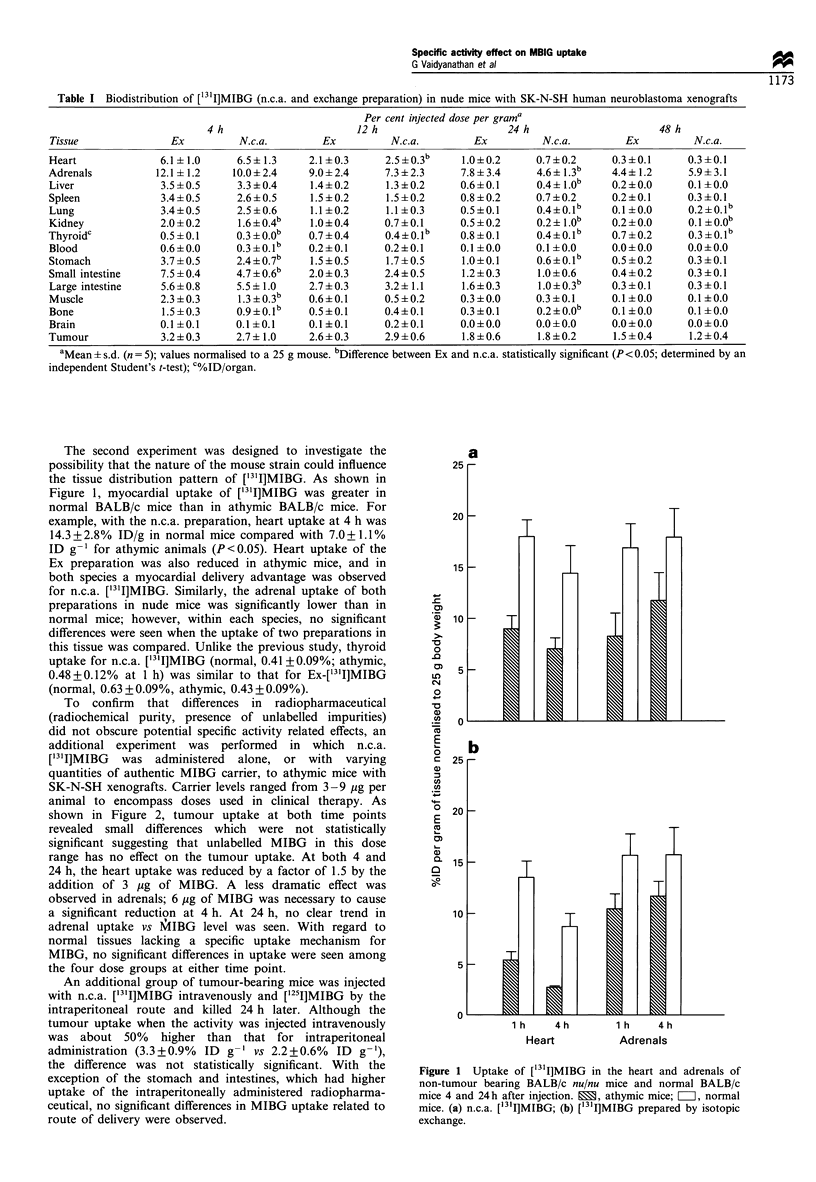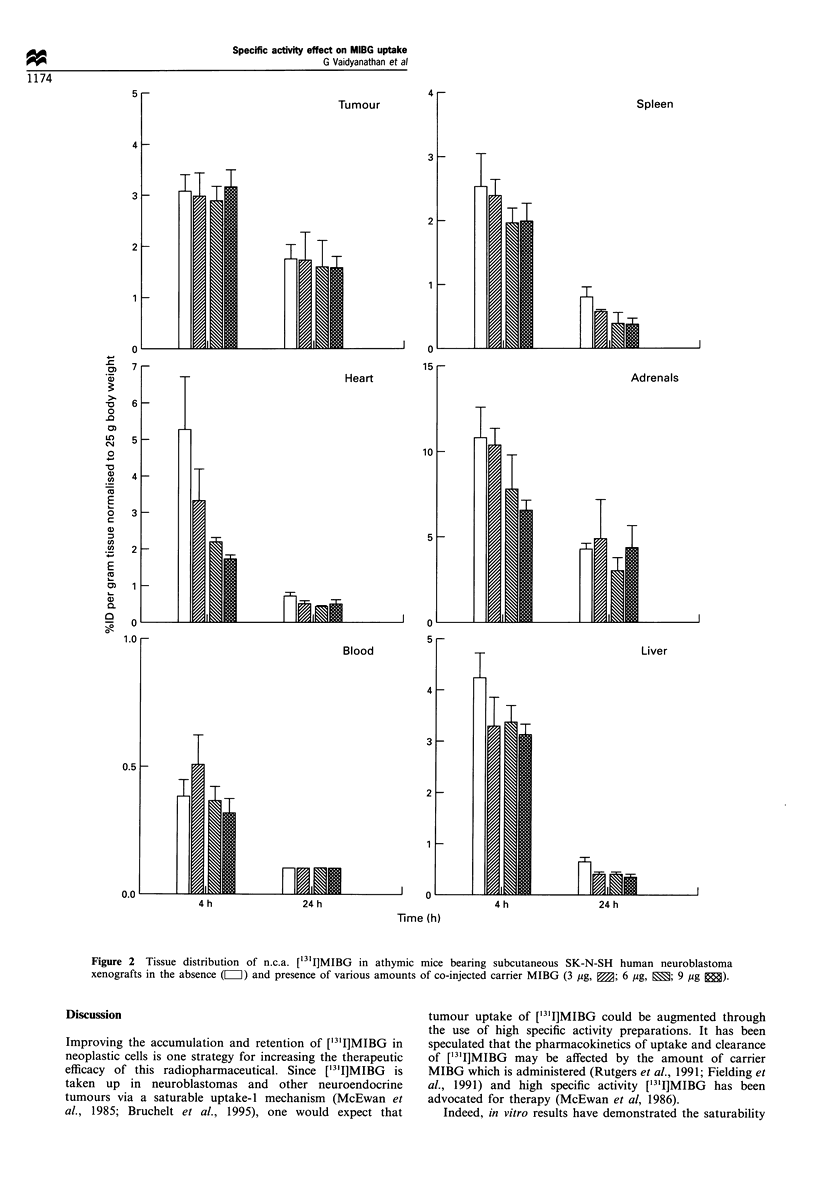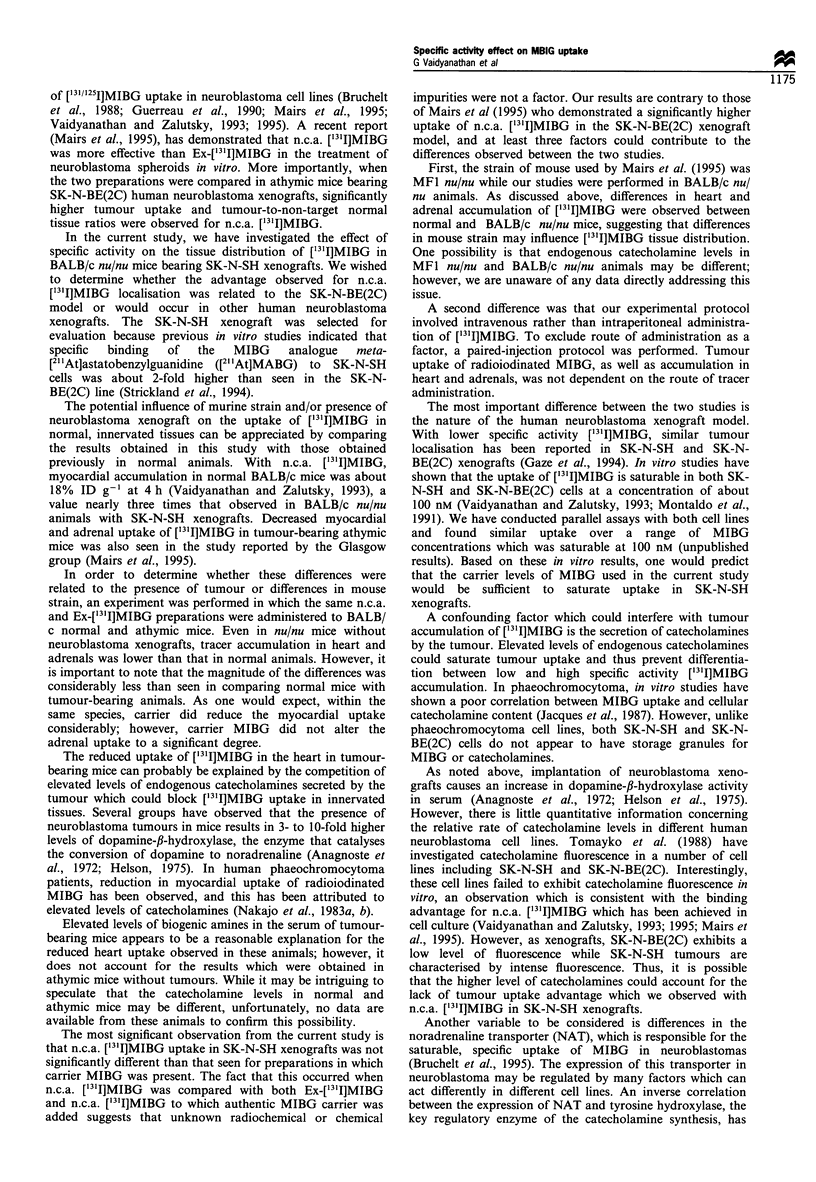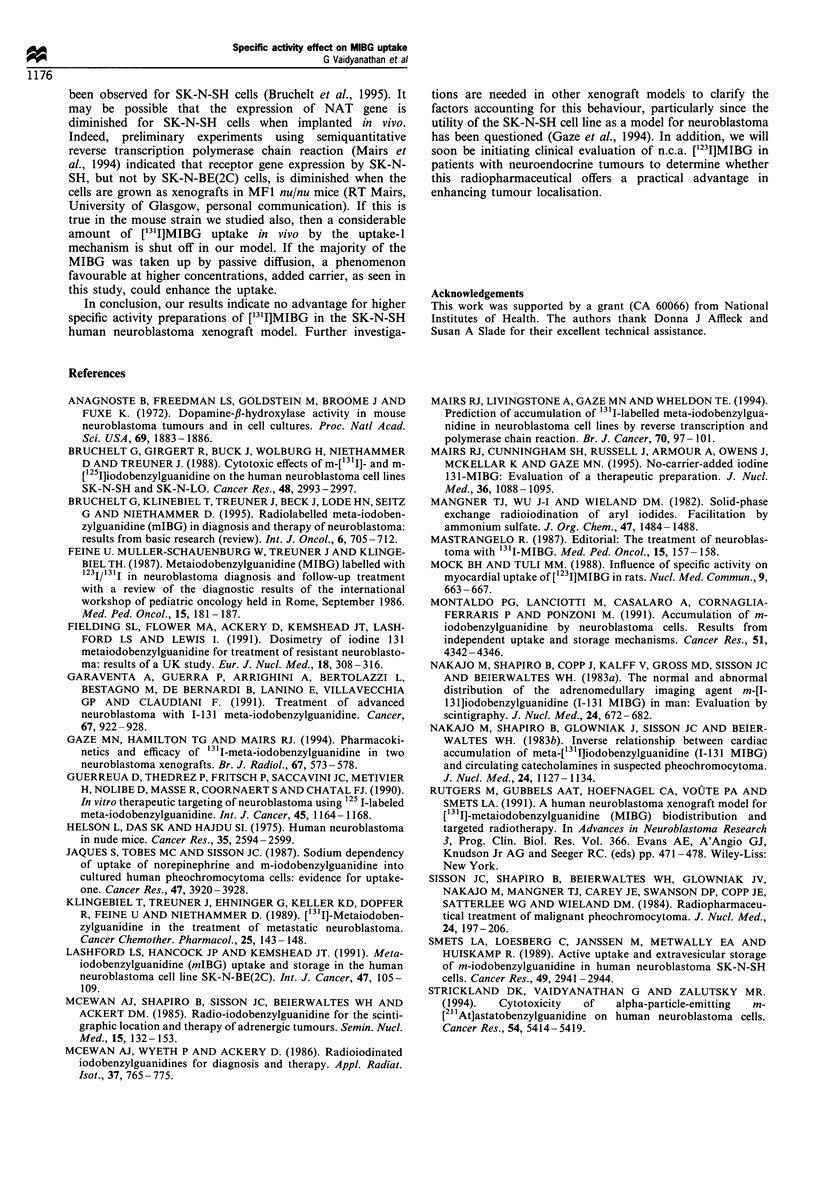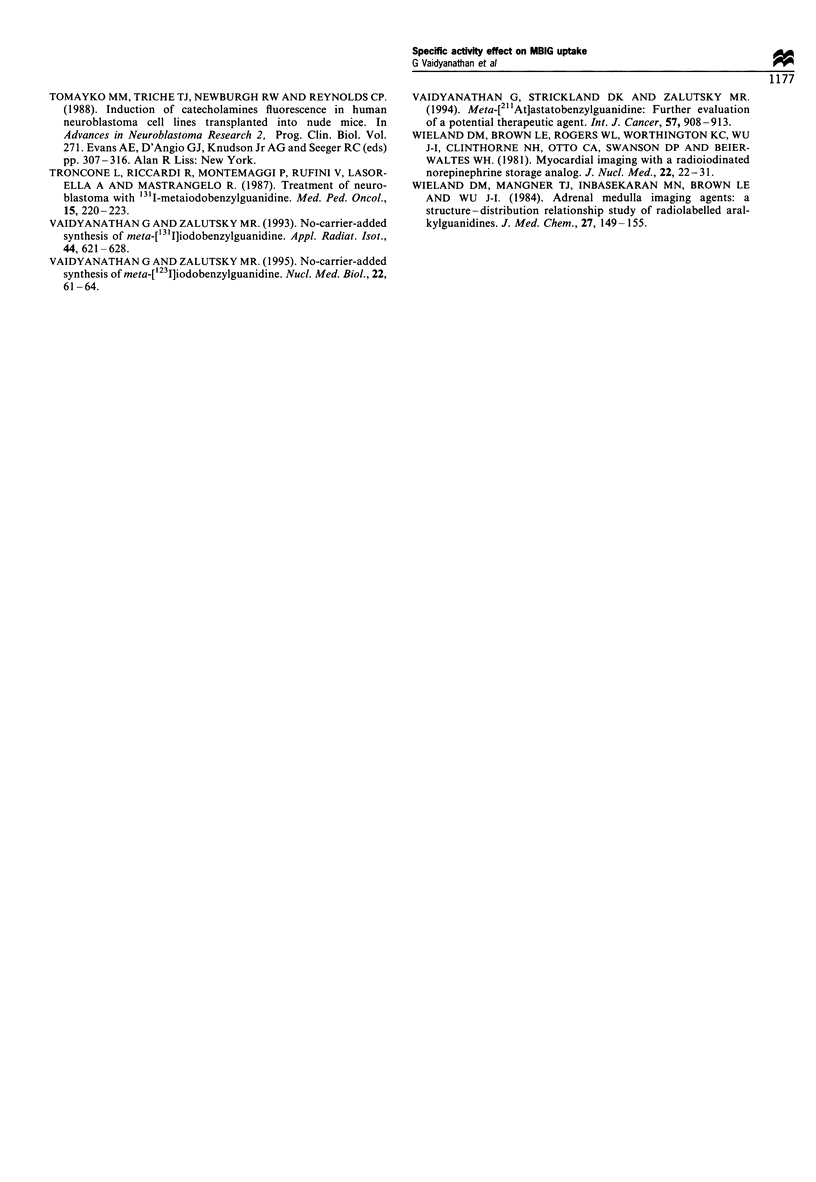# Localisation of [131I]MIBG in nude mice bearing SK-N-SH human neuroblastoma xenografts: effect of specific activity.

**DOI:** 10.1038/bjc.1996.226

**Published:** 1996-05

**Authors:** G. Vaidyanathan, H. S. Friedman, S. T. Keir, M. R. Zalutsky

**Affiliations:** Department of Radiology, Duke University Medical Center, Durham, North Carolina 27710, USA.

## Abstract

The biodistribution of no-carrier-added (n.c.a.) meta-[131I]iodobenzylguanidine ([131I]MIBG) and that prepared by the standard isotopic exchange method were compared in athymic mice bearing SK-N-SH human neuroblastoma xenografts. No advantage in tumour uptake was observed for the n.c.a. preparation. BALB/c nu/nu mice exhibited lower uptake in highly innervated normal tissues (heart and adrenals) than normal BALB/c mice. In another experiment, the distribution of n.c.a. [131I]MIBG in the absence or presence (3-9 micrograms) of MIBG carrier was determined. At both 4 h and 24 h, the heart uptake was reduced by a factor of 1.5 even at a dose of 3 micrograms MIBG. Tumour uptake was not significantly altered by various amounts of unlabelled MIBG at either time point.


British Journal of Cancer (1996) 73, 1171-1177
© 1996 Stockton Press All rights reserved 0007-0920/96 $12.00

### Localisation of [$^{131}$I]MIBG in nude mice bearing SK-N-SH human neuroblastoma xenografts: effect of specific activity

G Vaidyanathan[1], HS Friedman[2,3], ST Keir[2] and MR Zalutsky[3]

*Departments of [1]Radiology, [2]Paediatrics and [3]Pathology, Duke University Medical Center, Durham, North Carolina, USA.*

**Summary** The biodistribution of no-carrier-added (n.c.a) *meta*-[$^{131}$I]iodobenzylguanidine ([$^{131}$I]MIBG) and that prepared by the standard isotopic exchange method were compared in athymic mice bearing SK-N-SH human neuroblastoma xenografts. No advantage in tumour uptake was observed for the n.c.a. preparation. BALB/*c nu*/*nu* mice exhibited lower uptake in highly innervated normal tissues (heart and adrenals) than normal BALB/c mice. In another experiment, the distribution of n.c.a. [$^{131}$I]MIBG in the absence or presence (3–9 $\mu$g) of MIBG carrier was determined. At both 4 h and 24 h, the heart uptake was reduced by a factor of 1.5 even at a dose of 3 $\mu$g MIBG. Tumour uptake was not significantly altered by various amounts of unlabelled MIBG at either time point.

**Keywords:** neuroblastoma; *meta*-iodobenzylguanidine; specific activity

Radioiodinated *meta*-iodobenzylguanidine (MIBG), which is an analogue of noradrenaline has found widespread application in the management of neuroendocrine tumours such as phaeochromocytoma and neuroblastoma (Feine *et al.*, 1987; McEwan *et al.*, 1985; Sisson *et al.*, 1984; Troncone *et al.*, 1987). With regard to its diagnostic use, [$^{123}$I]MIBG and [$^{131}$I]MIBG imaging has been found to be quite sensitive and specific for lesion localisation. For example, in the case of neuroblastoma, false-positive scans are virtually non-existent and the number of false-negative scans is generally low (Mastrangelo, 1987). Iodine-131-labelled MIBG is under active investigation for the targeted radiotherapy of neuroblastoma (Klingebiel *et al.*, 1989; Garaventa *et al.*, 1991). The responses observed in some patients have been highly encouraging; however, it is clear that improvements in this treatment approach are needed.

One factor which could affect the efficacy of [$^{131}$I]MIBG is the specific activity of the radiopharmaceutical. Radioiodinated MIBG, as currently used in the clinic, is prepared by an isotopic exchange method (Ex-[$^{131}$I]MIBG) (Mangner *et al.*, 1982). This method yields preparations with a maximum specific activity of the order of 100 Ci mmol$^{-1}$ and hence includes significant levels of carrier MIBG. For example, a typical therapy dose of [$^{131}$I]MIBG contains about 200 $\mu$g of carrier MIBG per kg of body weight (Fielding *et al.*, 1991).

As MIBG is taken up in neuroblastoma cells *in vitro* and innervated tissues such as heart and adrenals *in vivo* primarily by a saturable mechanism (Wieland *et al.*, 1981; Smets *et al.*, 1989; Jaques *et al.*, 1987), one would expect that increasing the specific activity of radioiodinated MIBG could lead to an increase in tracer accumulation in target tissues. However, conflicting results on the effect of specific activity on MIBG distribution have been reported. For example, high and relatively constant myocardial uptake of [$^{125}$I]MIBG was reported over a wide range of specific activity; however, the greatest heart accumulation was observed with the lowest specific activity (Wieland *et al.*, 1981); similar behaviour was seen in the adrenals (Wieland *et al.*, 1984). On the contrary, saturation of heart uptake with low specific activity [$^{123}$I]MIBG also has been reported in a subsequent study (Mock and Tuli, 1988).

A number of laboratories have investigated the effect of specific-activity on the interaction of MIBG with tumour cells. *In vitro* studies using SK-N-SH human neuroblastoma cells have demonstrated that high specific activity [$^{131}$I]MIBG was more cytotoxic than a low specific activity preparation at the same radioactivity dose level (Bruchelt *et al.*, 1988). However, in a preliminary report no significant difference in tumour uptake was observed *in vivo* between [$^{131}$I]MIBG preparations of high and low specific activities in mice bearing SK-N-SH xenografts (Rutgers *et al.*, 1991).

Recently, we have developed a no-carrier-added (n.c.a.) synthesis of radioiodinated MIBG (Vaidyanathan and Zalutsky, 1993, 1995). With the availability of this labelling chemistry, investigations of the effect of specific activity on MIBG accumulation can now be extended to the n.c.a. level. We and others have shown that *in vitro*, the specific binding of n.c.a. [$^{131/125}$I]MIBG to human neuroblastoma cell lines remained constant over a 2-3-log activity range while that of Ex-[$^{131/125}$I]MIBG dropped by a factor of 7 (Vaidyanathan and Zalutsky, 1993; 1995; Mairs *et al.*, 1995), suggesting a lack of saturability for the n.c.a. preparation. The myocardial and adrenal uptake of the n.c.a. preparation was also found to be significantly higher than that observed for Ex-[$^{131}$I]MIBG. Furthermore, about an order of magnitude higher activity levels of Ex-[$^{131}$I]MIBG were necessary to have the same cytotoxic effect as seen with n.c.a. [$^{131}$I]MIBG (Vaidyanathan *et al.*, 1994).

Recently, the tissue distribution of n.c.a. [$^{131}$I]MIBG and a commercially available lot of this tracer prepared by the exchange reaction have been compared in mice bearing SK-N-BE(2C) human neuroblastoma xenografts (Mairs *et al.*, 1995). Highly encouraging results were obtained; the accumulation of n.c.a. [$^{131}$I]MIBG was significantly higher in tumour (as well as other target tissues such as heart and adrenals) and more favourable tumour-to-non-target normal tissue ratios were also seen for n.c.a. [$^{131}$I]MIBG.

The current study was undertaken to determine whether n.c.a. [$^{131}$I]MIBG would also offer a significant advantage in tumour accumulation in another human neuroblastoma xenograft model. The SK-N-SH xenograft model was selected for these experiments because a previous investigation in this model failed to show an increase in tumour localisation with increasing specific activity (Rutgers *et al.*, 1991). Our results suggest that the strain of the animal and the nature of the tumour line must be considered in interpreting specific activity effects on MIBG distribution.

### Materials and methods

### *General*

Radioiodine was obtained as a sodium [$^{131}$I]iodide or sodium [$^{125}$I]iodide solution in 0.1 N sodium hydroxide (NaOH) from

Correspondence: G Vaidyanathan, Box 3808, Department of Radiology, Duke University Medical Center, Durham, North Carolina 27710, USA.
Received 27 September 1995; revised 4 December 1995; accepted 7 December 1995

DuPont-New England Nuclear (North Billerica, MA, USA). *Meta*-iodobenzylguanidine hemisulphate was purchased from Sigma (St Louis, MO, USA). SK-N-SH human neuroblastoma cells were obtained from American Type Culture Collection (Rockville, MD, USA). The tissue culture medium (JRH Biosciences, Lenexa, KS, USA) was made by mixing 440 ml of RPMI-1640, 50 ml of Serum Plus, 5 ml of penicillin-G/streptomycin (5000 U of penicillin and 5000 $\mu$g of streptomycin per ml) and 5 ml of glutamine (200 mM in saline). The cells were grown at 37°C in a humidified incubator in a 5% carbon dioxide atmosphere.

### *Preparation of n.c.a [$^{131/125}$I]MIBG*

No-carrier-added [$^{131}$I]MIBG was prepared from 3-(trimethylsilylbenzylguanidine (TMSBG) as reported previously (Vaidyanathan and Zalutsky, 1993). Briefly, to the required amount of sodium [$^{131}$I]iodide in 1–3 $\mu$l of 0.1N NaOH was added 10 $\mu$l of a 0.3 M solution of *N*-chlorosuccinimide in trifluoroacetic acid, followed by TMSBG in trifluoroacetic acid (5 $\mu$l of 0.1 M solution). After 5 min at room temperature, the product was isolated by reverse-phase high pressure liquid chromatography (HPLC) in more than 90% radiochemical yield. The HPLC fractions containing [$^{131}$I]MIBG were pooled and most of the tetrahydrofuran present in the HPLC eluent was removed with a stream of argon and the remaining solution was passed through an activated $C_{18}$ solid-phase cartridge (Waters). After washing with 2 × 5 ml of water, 1 ml of 5 mM acetate buffer (pH 4.5) was passed through the cartridge. The activity was subsequently eluted with four 250 $\mu$l portions of methanol. The methanol portions containing the n.c.a. [$^{131}$I]MIBG were pooled, evaporated to dryness and reconstituted in phosphate-buffered saline (PBS). The same protocol was followed for the preparation of n.c.a. [$^{125}$I]MIBG.

Ex-[$^{131}$I]MIBG was prepared by the standard method (Mangner *et al.*, 1982) at the Duke University Radiopharmacy and was supplied as a solution in 5 mM acetate buffer (pH 4.5). The specific activity was 4–5 mCi (148–185 MBq) per mg and the activity concentration was about 5 mCi (185 MBq) ml$^{-1}$. The preparation was diluted in PBS before injection.

No-carrier-added [$^{131}$I]MIBG was prepared just before each tissue distribution experiment and was isolated by HPLC; the radiochemical purity was generally more than 99%. The purity of Ex-[$^{131}$I]MIBG, determined by HPLC, was more than 95%.

### *Experimental animals*

Athymic mice were obtained from a closed breeding colony maintained at the Duke University Cancer Center Isolation Facility. Six-week-old BALB/c *nu*/*nu* mice weighing 18–22 g were used in this study. In one experiment, normal male BALB/c mice weighing 25–28 g were used. All animal experimentation was carried out in accordance with a protocol approved by the Duke University Institutional Animal Care and Use Committee.

### *Xenograft model*

After testing for mycoplasma, cells were grown as monolayers in the tissue culture media described above. Cells ($10^8$ per ml) in serum-free medium were initially injected subcutaneously in the right flank of a few mice and tumours were allowed to grow for about 30 days. The tumours were isolated, homogenised and 100 $\mu$l of homogenate was injected into mice. Tissue distribution studies were performed about 30 days later when tumours had reached a volume of about 500–1000 mm$^3$.

### *Biodistribution measurements*

A total of four experiments were performed. In all experiments, groups of five mice were used for each time point. At selected intervals mice were killed with an overdose of halothane, dissected and tissues of interest were removed. After washing with saline, blot-drying and weighing, the tissues were counted for $^{131}$I activity in an automated gamma counter along with injection standards. The percentage of the injected dose per gram of tissue (%ID g$^{-1}$) was calculated for all tissues of interest using an in-house computer program. Although there were no significant differences between the organ weights of normal and nude mice, these values (from first three experiments; a paired-label protocol was used for the last experiment) were normalised to a 25 g mouse to minimise any influence the differential organ weights and blood volume may have on the tissue uptake. Tumour sizes in all groups within each experiment were not significantly different. An independent Student's *t*-test was used to determine statistical significance of differences observed between experimental groups. A $P < 0.05$ was considered to be statistically significant.

In the first experiment, athymic mice bearing subcutaneous SK-N-SH xenografts were injected intravenously with either 7 $\mu$Ci (0.3 MBq; containing 2 $\mu$g of carrier MIBG) of Ex-[$^{131}$I]MIBG or 11 $\mu$Ci (0.4 MBq) of n.c.a. [$^{131}$I]MIBG. Groups of animals were killed at 4, 12, 24 and 48 h post-injection and the tissue distribution of $^{131}$I activity was determined. A second experiment was performed to determine whether the mouse strain influenced tracer distribution. Both athymic mice (without xenografts) and normal BALB/c mice were injected intravenously with 12 $\mu$Ci of either Ex-[$^{131}$I]MIBG (0.4 MBq; 2.3 $\mu$g of MIBG) or n.c.a. [$^{131}$I]MIBG and the tissue distribution was determined at 1 and 4 h post-injection. In the third experiment, 5 $\mu$Ci (0.2 MBq) of n.c.a. [$^{131}$I]MIBG was administered to athymic mice with SK-N-SH xenografts with or without carrier MIBG (3, 6 or 9 $\mu$g per mouse) and the tissue uptake was determined at 4 and 24 h post-injection. Finally, to determine whether route of administration influenced tissue distribution, a paired-injection protocol was performed; five mice were injected with 6 $\mu$Ci (0.2 MBq) of n.c.a. [$^{131}$I]MIBG via the tail vein and 7 $\mu$Ci (0.3 MBq) of n.c.a. [$^{125}$I]MIBG was given by the intraperitoneal route. The tissue distribution of both radionuclides was determined at 24 h post-injection using a dual-channel gamma-counter.

### Results

The first biodistribution experiment was performed to compare the target and non-target organ uptake of n.c.a. [$^{131}$I]MIBG with that of an exchange preparation containing 2 $\mu$g of MIBG carrier. As summarised in Table I, both preparations were characterised by a rapid tissue clearance of $^{131}$I activity. Selective targeting in highly innervated normal tissues, i.e. heart and adrenals, was observed; however, the %ID g$^{-1}$ levels were less than anticipated based on previous studies in normal mice. In general, no clear advantage was seen for the n.c.a. preparation. Except the 12 h time point, the heart uptake was similar for both preparations. The heart uptake of n.c.a. [$^{131}$I]MIBG at 12 h (2.5 ± 0.3%ID g$^{-1}$) was slightly higher than that of exchange preparation (2.1 ± 0.3%ID g$^{-1}$) and the difference was statistically significant ($P < 0.05$). Adrenal uptake was also similar for both preparations except at 24 h, when that of Ex-[$^{131}$I]MIBG was slightly higher ($P < 0.05$). Tumour weights determined at necropsy for the animals receiving n.c.a. [$^{131}$]MIBG were not significantly different than those receiving Ex-[$^{131}$I]MIBG. For example, in the 4 h post-injection groups, tumour weights were 0.55 ± 0.29 g and 0.51 ± 0.29 g, for the n.c.a. and Ex groups, respectively. Accumulation in SK-N-SH xenografts remained constant for the first 12 h and then declined gradually thereafter. No statistically significant advantage in tumour uptake was observed for the n.c.a. preparation. Thyroid accumulation of n.c.a. [$^{131}$I]MIBG was consistently 2–3 times lower than that of the exchange preparation, suggesting more favourable radiochemical purity, stability or a greater inertness to deiodination *in vivo*.

**Table I** Biodistribution of [$^{131}$I]MIBG (n.c.a. and exchange preparation) in nude mice with SK-N-SH human neuroblastoma xenografts

| | *Per cent injected dose per gram*[a] | | | | | | | |
|---|---|---|---|---|---|---|---|---|
| | *4 h* | | *12 h* | | *24 h* | | *48 h* | |
| *Tissue* | *Ex* | *N.c.a.* | *Ex* | *N.c.a.* | *Ex* | *N.c.a.* | *Ex* | *N.c.a.* |
| Heart | 6.1 ± 1.0 | 6.5 ± 1.3 | 2.1 ± 0.3 | 2.5 ± 0.3[b] | 1.0 ± 0.2 | 0.7 ± 0.2 | 0.3 ± 0.1 | 0.3 ± 0.1 |
| Adrenals | 12.1 ± 1.2 | 10.0 ± 2.4 | 9.0 ± 2.4 | 7.3 ± 2.3 | 7.8 ± 3.4 | 4.6 ± 1.3[b] | 4.4 ± 1.2 | 5.9 ± 3.1 |
| Liver | 3.5 ± 0.5 | 3.3 ± 0.4 | 1.4 ± 0.2 | 1.3 ± 0.2 | 0.6 ± 0.1 | 0.4 ± 1.0[b] | 0.2 ± 0.0 | 0.1 ± 0.0 |
| Spleen | 3.4 ± 0.5 | 2.6 ± 0.5 | 1.5 ± 0.2 | 1.5 ± 0.2 | 0.8 ± 0.2 | 0.7 ± 0.2 | 0.2 ± 0.1 | 0.3 ± 0.1 |
| Lung | 3.4 ± 0.5 | 2.5 ± 0.6 | 1.1 ± 0.2 | 1.1 ± 0.3 | 0.5 ± 0.1 | 0.4 ± 0.1[b] | 0.1 ± 0.0 | 0.2 ± 0.1[b] |
| Kidney | 2.0 ± 0.2 | 1.6 ± 0.4[b] | 1.0 ± 0.4 | 0.7 ± 0.1 | 0.5 ± 0.2 | 0.2 ± 1.0[b] | 0.2 ± 0.0 | 0.1 ± 0.0[b] |
| Thyroid[c] | 0.5 ± 0.1 | 0.3 ± 0.0[b] | 0.7 ± 0.4 | 0.4 ± 0.1[b] | 0.8 ± 0.1 | 0.4 ± 0.1[b] | 0.7 ± 0.2 | 0.3 ± 0.1[b] |
| Blood | 0.6 ± 0.0 | 0.3 ± 0.1[b] | 0.2 ± 0.1 | 0.2 ± 0.1 | 0.1 ± 0.0 | 0.1 ± 0.0 | 0.0 ± 0.0 | 0.0 ± 0.0 |
| Stomach | 3.7 ± 0.5 | 2.4 ± 0.7[b] | 1.5 ± 0.5 | 1.7 ± 0.5 | 1.0 ± 0.1 | 0.6 ± 0.1[b] | 0.5 ± 0.2 | 0.3 ± 0.1 |
| Small intestine | 7.5 ± 0.4 | 4.7 ± 0.6[b] | 2.0 ± 0.3 | 2.4 ± 0.5 | 1.2 ± 0.3 | 1.0 ± 0.6 | 0.4 ± 0.2 | 0.3 ± 0.1 |
| Large intestine | 5.6 ± 0.8 | 5.5 ± 1.0 | 2.7 ± 0.3 | 3.2 ± 1.1 | 1.6 ± 0.3 | 1.0 ± 0.3[b] | 0.3 ± 0.1 | 0.3 ± 0.1 |
| Muscle | 2.3 ± 0.3 | 1.3 ± 0.3[b] | 0.6 ± 0.1 | 0.5 ± 0.2 | 0.3 ± 0.0 | 0.3 ± 0.1 | 0.1 ± 0.0 | 0.1 ± 0.0 |
| Bone | 1.5 ± 0.3 | 0.9 ± 0.1[b] | 0.5 ± 0.1 | 0.4 ± 0.1 | 0.3 ± 0.1 | 0.2 ± 0.0[b] | 0.1 ± 0.0 | 0.1 ± 0.0 |
| Brain | 0.1 ± 0.1 | 0.1 ± 0.1 | 0.1 ± 0.1 | 0.2 ± 0.1 | 0.0 ± 0.0 | 0.0 ± 0.0 | 0.0 ± 0.0 | 0.0 ± 0.0 |
| Tumour | 3.2 ± 0.3 | 2.7 ± 1.0 | 2.6 ± 0.3 | 2.9 ± 0.6 | 1.8 ± 0.6 | 1.8 ± 0.2 | 1.5 ± 0.4 | 1.2 ± 0.4 |

[a]Mean ± s.d. ($n = 5$); values normalised to a 25 g mouse. [b]Difference between Ex and n.c.a. statistically significant ($P < 0.05$; determined by an independent Student's $t$-test); [c]%ID/organ.

The second experiment was designed to investigate the possibility that the nature of the mouse strain could influence the tissue distribution pattern of [$^{131}$I]MIBG. As shown in Figure 1, myocardial uptake of [$^{131}$I]MIBG was greater in normal BALB/c mice than in athymic BALB/c mice. For example, with the n.c.a. preparation, heart uptake at 4 h was 14.3 ± 2.8% ID/g in normal mice compared with 7.0 ± 1.1% ID $g^{-1}$ for athymic animals ($P < 0.05$). Heart uptake of the Ex preparation was also reduced in athymic mice, and in both species a myocardial delivery advantage was observed for n.c.a. [$^{131}$I]MIBG. Similarly, the adrenal uptake of both preparations in nude mice was significantly lower than in normal mice; however, within each species, no significant differences were seen when the uptake of two preparations in this tissue was compared. Unlike the previous study, thyroid uptake for n.c.a. [$^{131}$I]MIBG (normal, 0.41 ± 0.09%; athymic, 0.48 ± 0.12% at 1 h) was similar to that for Ex-[$^{131}$I]MIBG (normal, 0.63 ± 0.09%, athymic, 0.43 ± 0.09%).

To confirm that differences in radiopharmaceutical (radiochemical purity, presence of unlabelled impurities) did not obscure potential specific activity related effects, an additional experiment was performed in which n.c.a. [$^{131}$I]MIBG was administered alone, or with varying quantities of authentic MIBG carrier, to athymic mice with SK-N-SH xenografts. Carrier levels ranged from 3–9 μg per animal to encompass doses used in clinical therapy. As shown in Figure 2, tumour uptake at both time points revealed small differences which were not statistically significant suggesting that unlabelled MIBG in this dose range has no effect on the tumour uptake. At both 4 and 24 h, the heart uptake was reduced by a factor of 1.5 by the addition of 3 μg of MIBG. A less dramatic effect was observed in adrenals; 6 μg of MIBG was necessary to cause a significant reduction at 4 h. At 24 h, no clear trend in adrenal uptake *vs* MIBG level was seen. With regard to normal tissues lacking a specific uptake mechanism for MIBG, no significant differences in uptake were seen among the four dose groups at either time point.

An additional group of tumour-bearing mice was injected with n.c.a. [$^{131}$I]MIBG intravenously and [$^{125}$I]MIBG by the intraperitoneal route and killed 24 h later. Although the tumour uptake when the activity was injected intravenously was about 50% higher than that for intraperitoneal administration (3.3 ± 0.9% ID $g^{-1}$ *vs* 2.2 ± 0.6% ID $g^{-1}$), the difference was not statistically significant. With the exception of the stomach and intestines, which had higher uptake of the intraperitoneally administered radiopharmaceutical, no significant differences in MIBG uptake related to route of delivery were observed.

**Figure 1** Uptake of [$^{131}$I]MIBG in the heart and adrenals of non-tumour bearing BALB/c *nu/nu* mice and normal BALB/c mice 4 and 24 h after injection. ▨, athymic mice; □, normal mice. (**a**) n.c.a. [$^{131}$I]MIBG; (**b**) [$^{131}$I]MIBG prepared by isotopic exchange.

**Figure 2** Tissue distribution of n.c.a. [$^{131}$I]MIBG in athymic mice bearing subcutaneous SK-N-SH human neuroblastoma xenografts in the absence (▭) and presence of various amounts of co-injected carrier MIBG (3 μg, ▨; 6 μg, ▧; 9 μg ▩).

### Discussion

Improving the accumulation and retention of [$^{131}$I]MIBG in neoplastic cells is one strategy for increasing the therapeutic efficacy of this radiopharmaceutical. Since [$^{131}$I]MIBG is taken up in neuroblastomas and other neuroendocrine tumours via a saturable uptake-1 mechanism (McEwan *et al.*, 1985; Bruchelt *et al.*, 1995), one would expect that tumour uptake of [$^{131}$I]MIBG could be augmented through the use of high specific activity preparations. It has been speculated that the pharmacokinetics of uptake and clearance of [$^{131}$I]MIBG may be affected by the amount of carrier MIBG which is administered (Rutgers *et al.*, 1991; Fielding *et al.*, 1991) and high specific activity [$^{131}$I]MIBG has been advocated for therapy (McEwan *et al*, 1986).

Indeed, *in vitro* results have demonstrated the saturability of [$^{131/125}$I]MIBG uptake in neuroblastoma cell lines (Bruchelt *et al.*, 1988; Guerreau *et al.*, 1990; Mairs *et al.*, 1995; Vaidyanathan and Zalutsky, 1993; 1995). A recent report (Mairs *et al.*, 1995), has demonstrated that n.c.a. [$^{131}$I]MIBG was more effective than Ex-[$^{131}$I]MIBG in the treatment of neuroblastoma spheroids *in vitro*. More importantly, when the two preparations were compared in athymic mice bearing SK-N-BE(2C) human neuroblastoma xenografts, significantly higher tumour uptake and tumour-to-non-target normal tissue ratios were observed for n.c.a. [$^{131}$I]MIBG.

In the current study, we have investigated the effect of specific activity on the tissue distribution of [$^{131}$I]MIBG in BALB/c *nu*/*nu* mice bearing SK-N-SH xenografts. We wished to determine whether the advantage observed for n.c.a. [$^{131}$I]MIBG localisation was related to the SK-N-BE(2C) model or would occur in other human neuroblastoma xenografts. The SK-N-SH xenograft was selected for evaluation because previous *in vitro* studies indicated that specific binding of the MIBG analogue *meta*-[$^{211}$At]astatobenzylguanidine ([$^{211}$At]MABG) to SK-N-SH cells was about 2-fold higher than seen in the SK-N-BE(2C) line (Strickland *et al.*, 1994).

The potential influence of murine strain and/or presence of neuroblastoma xenograft on the uptake of [$^{131}$I]MIBG in normal, innervated tissues can be appreciated by comparing the results obtained in this study with those obtained previously in normal animals. With n.c.a. [$^{131}$I]MIBG, myocardial accumulation in normal BALB/c mice was about 18% ID $g^{-1}$ at 4 h (Vaidyanathan and Zalutsky, 1993), a value nearly three times that observed in BALB/c *nu*/*nu* animals with SK-N-SH xenografts. Decreased myocardial and adrenal uptake of [$^{131}$I]MIBG in tumour-bearing athymic mice was also seen in the study reported by the Glasgow group (Mairs *et al.*, 1995).

In order to determine whether these differences were related to the presence of tumour or differences in mouse strain, an experiment was performed in which the same n.c.a. and Ex-[$^{131}$I]MIBG preparations were administered to BALB/c normal and athymic mice. Even in *nu*/*nu* mice without neuroblastoma xenografts, tracer accumulation in heart and adrenals was lower than that in normal animals. However, it is important to note that the magnitude of the differences was considerably less than seen in comparing normal mice with tumour-bearing animals. As one would expect, within the same species, carrier did reduce the myocardial uptake considerably; however, carrier MIBG did not alter the adrenal uptake to a significant degree.

The reduced uptake of [$^{131}$I]MIBG in the heart in tumour-bearing mice can probably be explained by the competition of elevated levels of endogenous catecholamines secreted by the tumour which could block [$^{131}$I]MIBG uptake in innervated tissues. Several groups have observed that the presence of neuroblastoma tumours in mice results in 3- to 10-fold higher levels of dopamine-$\beta$-hydroxylase, the enzyme that catalyses the conversion of dopamine to noradrenaline (Anagnoste *et al.*, 1972; Helson, 1975). In human phaeochromocytoma patients, reduction in myocardial uptake of radioiodinated MIBG has been observed, and this has been attributed to elevated levels of catecholamines (Nakajo *et al.*, 1983*a*, *b*).

Elevated levels of biogenic amines in the serum of tumour-bearing mice appears to be a reasonable explanation for the reduced heart uptake observed in these animals; however, it does not account for the results which were obtained in athymic mice without tumours. While it may be intriguing to speculate that the catecholamine levels in normal and athymic mice may be different, unfortunately, no data are available from these animals to confirm this possibility.

The most significant observation from the current study is that n.c.a. [$^{131}$I]MIBG uptake in SK-N-SH xenografts was not significantly different than that seen for preparations in which carrier MIBG was present. The fact that this occurred when n.c.a. [$^{131}$I]MIBG was compared with both Ex-[$^{131}$I]MIBG and n.c.a. [$^{131}$I]MIBG to which authentic MIBG carrier was added suggests that unknown radiochemical or chemical impurities were not a factor. Our results are contrary to those of Mairs *et al* (1995) who demonstrated a significantly higher uptake of n.c.a. [$^{131}$I]MIBG in the SK-N-BE(2C) xenograft model, and at least three factors could contribute to the differences observed between the two studies.

First, the strain of mouse used by Mairs *et al.* (1995) was MF1 *nu*/*nu* while our studies were performed in BALB/c *nu*/*nu* animals. As discussed above, differences in heart and adrenal accumulation of [$^{131}$I]MIBG were observed between normal and BALB/c *nu*/*nu* mice, suggesting that differences in mouse strain may influence [$^{131}$I]MIBG tissue distribution. One possibility is that endogenous catecholamine levels in MF1 *nu*/*nu* and BALB/c *nu*/*nu* animals may be different; however, we are unaware of any data directly addressing this issue.

A second difference was that our experimental protocol involved intravenous rather than intraperitoneal administration of [$^{131}$I]MIBG. To exclude route of administration as a factor, a paired-injection protocol was performed. Tumour uptake of radioiodinated MIBG, as well as accumulation in heart and adrenals, was not dependent on the route of tracer administration.

The most important difference between the two studies is the nature of the human neuroblastoma xenograft model. With lower specific activity [$^{131}$I]MIBG, similar tumour localisation has been reported in SK-N-SH and SK-N-BE(2C) xenografts (Gaze *et al.*, 1994). *In vitro* studies have shown that the uptake of [$^{131}$I]MIBG is saturable in both SK-N-SH and SK-N-BE(2C) cells at a concentration of about 100 nM (Vaidyanathan and Zalutsky, 1993; Montaldo *et al.*, 1991). We have conducted parallel assays with both cell lines and found similar uptake over a range of MIBG concentrations which was saturable at 100 nM (unpublished results). Based on these *in vitro* results, one would predict that the carrier levels of MIBG used in the current study would be sufficient to saturate uptake in SK-N-SH xenografts.

A confounding factor which could interfere with tumour accumulation of [$^{131}$I]MIBG is the secretion of catecholamines by the tumour. Elevated levels of endogenous catecholamines could saturate tumour uptake and thus prevent differentiation between low and high specific activity [$^{131}$I]MIBG accumulation. In phaeochromocytoma, *in vitro* studies have shown a poor correlation between MIBG uptake and cellular catecholamine content (Jacques *et al.*, 1987). However, unlike phaeochromocytoma cell lines, both SK-N-SH and SK-N-BE(2C) cells do not appear to have storage granules for MIBG or catecholamines.

As noted above, implantation of neuroblastoma xenografts causes an increase in dopamine-$\beta$-hydroxylase activity in serum (Anagnoste *et al.*, 1972; Helson *et al.*, 1975). However, there is little quantitative information concerning the relative rate of catecholamine levels in different human neuroblastoma cell lines. Tomayko *et al.* (1988) have investigated catecholamine fluorescence in a number of cell lines including SK-N-SH and SK-N-BE(2C). Interestingly, these cell lines failed to exhibit catecholamine fluorescence *in vitro*, an observation which is consistent with the binding advantage for n.c.a. [$^{131}$I]MIBG which has been achieved in cell culture (Vaidyanathan and Zalutsky, 1993; 1995; Mairs *et al.*, 1995). However, as xenografts, SK-N-BE(2C) exhibits a low level of fluorescence while SK-N-SH tumours are characterised by intense fluorescence. Thus, it is possible that the higher level of catecholamines could account for the lack of tumour uptake advantage which we observed with n.c.a. [$^{131}$I]MIBG in SK-N-SH xenografts.

Another variable to be considered is differences in the noradrenaline transporter (NAT), which is responsible for the saturable, specific uptake of MIBG in neuroblastomas (Bruchelt *et al.*, 1995). The expression of this transporter in neuroblastoma may be regulated by many factors which can act differently in different cell lines. An inverse correlation between the expression of NAT and tyrosine hydroxylase, the key regulatory enzyme of the catecholamine synthesis, has been observed for SK-N-SH cells (Bruchelt et al., 1995). It may be possible that the expression of NAT gene is diminished for SK-N-SH cells when implanted *in vivo*. Indeed, preliminary experiments using semiquantitative reverse transcription polymerase chain reaction (Mairs et al., 1994) indicated that receptor gene expression by SK-N-SH, but not by SK-N-BE(2C) cells, is diminished when the cells are grown as xenografts in MF1 *nu/nu* mice (RT Mairs, University of Glasgow, personal communication). If this is true in the mouse strain we studied also, then a considerable amount of [$^{131}$I]MIBG uptake *in vivo* by the uptake-1 mechanism is shut off in our model. If the majority of the MIBG was taken up by passive diffusion, a phenomenon favourable at higher concentrations, added carrier, as seen in this study, could enhance the uptake.

In conclusion, our results indicate no advantage for higher specific activity preparations of [$^{131}$I]MIBG in the SK-N-SH human neuroblastoma xenograft model. Further investigations are needed in other xenograft models to clarify the factors accounting for this behaviour, particularly since the utility of the SK-N-SH cell line as a model for neuroblastoma has been questioned (Gaze et al., 1994). In addition, we will soon be initiating clinical evaluation of n.c.a. [$^{123}$I]MIBG in patients with neuroendocrine tumours to determine whether this radiopharmaceutical offers a practical advantage in enhancing tumour localisation.

**Acknowledgements**
This work was supported by a grant (CA 60066) from National Institutes of Health. The authors thank Donna J Affleck and Susan A Slade for their excellent technical assistance.

### References

ANAGNOSTE B, FREEDMAN LS, GOLDSTEIN M, BROOME J AND FUXE K. (1972). Dopamine-$\beta$-hydroxylase activity in mouse neuroblastoma tumours and in cell cultures. *Proc. Natl Acad. Sci. USA*, **69**, 1883-1886.

BRUCHELT G, GIRGERT R, BUCK J, WOLBURG H, NIETHAMMER D AND TREUNER J. (1988). Cytotoxic effects of m-[$^{131}$I]- and m-[$^{125}$I]iodobenzylguanidine on the human neuroblastoma cell lines SK-N-SH and SK-N-LO. *Cancer Res.*, **48**, 2993-2997.

BRUCHELT G, KLINEBIEL T, TREUNER J, BECK J, LODE HN, SEITZ G AND NIETHAMMER D. (1995). Radiolabelled meta-iodobenzylguanidine (mIBG) in diagnosis and therapy of neuroblastoma: results from basic research (review). *Int. J. Oncol.*, **6**, 705-712.

FEINE U. MULLER-SCHAUENBURG W, TREUNER J AND KLINGEBIEL TH. (1987). Metaiodobenzylguanidine (MIBG) labelled with $^{123}$I/$^{131}$I in neuroblastoma diagnosis and follow-up treatment with a review of the diagnostic results of the international workshop of pediatric oncology held in Rome, September 1986. *Med. Ped. Oncol.*, **15**, 181-187.

FIELDING SL, FLOWER MA, ACKERY D, KEMSHEAD JT, LASHFORD LS AND LEWIS I. (1991). Dosimetry of iodine 131 metaiodobenzylguanidine for treatment of resistant neuroblastoma: results of a UK study. *Eur. J. Nucl. Med.*, **18**, 308-316.

GARAVENTA A, GUERRA P, ARRIGHINI A, BERTOLAZZI L, BESTAGNO M, DE BERNARDI B, LANINO E, VILLAVECCHIA GP AND CLAUDIANI F. (1991). Treatment of advanced neuroblastoma with I-131 meta-iodobenzylguanidine. *Cancer*, **67**, 922-928.

GAZE MN, HAMILTON TG AND MAIRS RJ. (1994). Pharmacokinetics and efficacy of $^{131}$I-meta-iodobenzylguanidine in two neuroblastoma xenografts. *Br. J. Radiol.*, **67**, 573-578.

GUERREUA D, THEDREZ P, FRITSCH P, SACCAVINI JC, METIVIER H, NOLIBE D, MASSE R, COORNAERT S AND CHATAL FJ. (1990). *In vitro* therapeutic targeting of neuroblastoma using $^{125}$ I-labeled meta-iodobenzylguanidine. *Int. J. Cancer*, **45**, 1164-1168.

HELSON L, DAS SK AND HAJDU SI. (1975). Human neuroblastoma in nude mice. *Cancer Res.*, **35**, 2594-2599.

JAQUES S, TOBES MC AND SISSON JC. (1987). Sodium dependency of uptake of norepinephrine and m-iodobenzylguanidine into cultured human pheochromocytoma cells: evidence for uptake-one. *Cancer Res.*, **47**, 3920-3928.

KLINGEBIEL T, TREUNER J, EHNINGER G, KELLER KD, DOPFER R, FEINE U AND NIETHAMMER D. (1989). [$^{131}$I]-Metaiodobenzylguanidine in the treatment of metastatic neuroblastoma. *Cancer Chemother. Pharmacol.*, **25**, 143-148.

LASHFORD LS, HANCOCK JP AND KEMSHEAD JT. (1991). *Meta*-iodobenzylguanidine (*m*IBG) uptake and storage in the human neuroblastoma cell line SK-N-BE(2C). *Int. J. Cancer*, **47**, 105-109.

MCEWAN AJ, SHAPIRO B, SISSON JC, BEIERWALTES WH AND ACKERT DM. (1985). Radio-iodobenzylguanidine for the scintigraphic location and therapy of adrenergic tumours. *Semin. Nucl. Med.*, **15**, 132-153.

MCEWAN AJ, WYETH P AND ACKERY D. (1986). Radioiodinated iodobenzylguanidines for diagnosis and therapy. *Appl. Radiat. Isot.*, **37**, 765-775.

MAIRS RJ, LIVINGSTONE A, GAZE MN AND WHELDON TE. (1994). Prediction of accumulation of $^{131}$I-labelled meta-iodobenzylguanidine in neuroblastoma cell lines by reverse transcription and polymerase chain reaction. *Br. J. Cancer*, **70**, 97-101.

MAIRS RJ, CUNNINGHAM SH, RUSSELL J, ARMOUR A, OWENS J, MCKELLAR K AND GAZE MN. (1995). No-carrier-added iodine 131-MIBG: Evaluation of a therapeutic preparation. *J. Nucl. Med.*, **36**, 1088-1095.

MANGNER TJ, WU J-I AND WIELAND DM. (1982). Solid-phase exchange radioiodination of aryl iodides. Facilitation by ammonium sulfate. *J. Org. Chem.*, **47**, 1484-1488.

MASTRANGELO R. (1987). Editorial: The treatment of neuroblastoma with $^{131}$I-MIBG. *Med. Ped. Oncol.*, **15**, 157-158.

MOCK BH AND TULI MM. (1988). Influence of specific activity on myocardial uptake of [$^{123}$I]MIBG in rats. *Nucl. Med. Commun.*, **9**, 663-667.

MONTALDO PG, LANCIOTTI M, CASALARO A, CORNAGLIA-FERRARIS P AND PONZONI M. (1991). Accumulation of *m*-iodobenzylguanidine by neuroblastoma cells. Results from independent uptake and storage mechanisms. *Cancer Res.*, **51**, 4342-4346.

NAKAJO M, SHAPIRO B, COPP J, KALFF V, GROSS MD, SISSON JC AND BEIERWALTES WH. (1983*a*). The normal and abnormal distribution of the adrenomedullary imaging agent *m*-[I-131]iodobenzylguanidine (I-131 MIBG) in man: Evaluation by scintigraphy. *J. Nucl. Med.*, **24**, 672-682.

NAKAJO M, SHAPIRO B, GLOWNIAK J, SISSON JC AND BEIERWALTES WH. (1983*b*). Inverse relationship between cardiac accumulation of meta-[$^{131}$I]iodobenzylguanidine (I-131 MIBG) and circulating catecholamines in suspected pheochromocytoma. *J. Nucl. Med.*, **24**, 1127-1134.

RUTGERS M, GUBBELS AAT, HOEFNAGEL CA, VOÛTE PA AND SMETS LA. (1991). A human neuroblastoma xenograft model for [$^{131}$I]-metaiodobenzylguanidine (MIBG) biodistribution and targeted radiotherapy. In *Advances in Neuroblastoma Research 3*, Prog. Clin. Biol. Res. Vol. 366. Evans AE, A'Angio GJ, Knudson Jr AG and Seeger RC. (eds) pp. 471-478. Wiley-Liss: New York.

SISSON JC, SHAPIRO B, BEIERWALTES WH, GLOWNIAK JV, NAKAJO M, MANGNER TJ, CAREY JE, SWANSON DP, COPP JE, SATTERLEE WG AND WIELAND DM. (1984). Radiopharmaceutical treatment of malignant pheochromocytoma. *J. Nucl. Med.*, **24**, 197-206.

SMETS LA, LOESBERG C, JANSSEN M, METWALLY EA AND HUISKAMP R. (1989). Active uptake and extravesicular storage of *m*-iodobenzylguanidine in human neuroblastoma SK-N-SH cells. *Cancer Res.*, **49**, 2941-2944.

STRICKLAND DK, VAIDYANATHAN G AND ZALUTSKY MR. (1994). Cytotoxicity of alpha-particle-emitting *m*-[$^{211}$At]astatobenzylguanidine on human neuroblastoma cells. *Cancer Res.*, **54**, 5414-5419.

TOMAYKO MM, TRICHE TJ, NEWBURGH RW AND REYNOLDS CP. (1988). Induction of catecholamines fluorescence in human neuroblastoma cell lines transplanted into nude mice. In *Advances in Neuroblastoma Research 2,* Prog. Clin. Biol. Vol. 271. Evans AE, D'Angio GJ, Knudson Jr AG and Seeger RC (eds) pp. 307-316. Alan R Liss: New York.

TRONCONE L, RICCARDI R, MONTEMAGGI P, RUFINI V, LASORELLA A AND MASTRANGELO R. (1987). Treatment of neuroblastoma with $^{131}$I-metaiodobenzylguanidine. *Med. Ped. Oncol.*, **15**, 220-223.

VAIDYANATHAN G AND ZALUTSKY MR. (1993). No-carrier-added synthesis of *meta*-[$^{131}$I]iodobenzylguanidine. *Appl. Radiat. Isot.*, **44**, 621-628.

VAIDYANATHAN G AND ZALUTSKY MR. (1995). No-carrier-added synthesis of *meta*-[$^{123}$I]iodobenzylguanidine. *Nucl. Med. Biol.*, **22**, 61-64.

VAIDYANATHAN G, STRICKLAND DK AND ZALUTSKY MR. (1994). *Meta*-[$^{211}$At]astatobenzylguanidine: Further evaluation of a potential therapeutic agent. *Int. J. Cancer*, **57**, 908-913.

WIELAND DM, BROWN LE, ROGERS WL, WORTHINGTON KC, WU J-I, CLINTHORNE NH, OTTO CA, SWANSON DP AND BEIERWALTES WH. (1981). Myocardial imaging with a radioiodinated norepinephrine storage analog. *J. Nucl. Med.*, **22**, 22-31.

WIELAND DM, MANGNER TJ, INBASEKARAN MN, BROWN LE AND WU J-I. (1984). Adrenal medulla imaging agents: a structure-distribution relationship study of radiolabelled aralkylguanidines. *J. Med. Chem.*, **27**, 149-155.